# Localized proteomic differences in the choroid plexus of Alzheimer's disease and epilepsy patients

**DOI:** 10.3389/fneur.2023.1221775

**Published:** 2023-07-14

**Authors:** Dominique F. Leitner, Evgeny Kanshin, Arline Faustin, Manon Thierry, Daniel Friedman, Sasha Devore, Beatrix Ueberheide, Orrin Devinsky, Thomas Wisniewski

**Affiliations:** ^1^Comprehensive Epilepsy Center, New York University Grossman School of Medicine, New York, NY, United States; ^2^Center for Cognitive Neurology, Department of Neurology, New York University Grossman School of Medicine, New York, NY, United States; ^3^Department of Neurology, New York University Grossman School of Medicine, New York, NY, United States; ^4^Proteomics Laboratory, Division of Advanced Research Technologies, New York University Grossman School of Medicine, New York, NY, United States; ^5^Department of Biochemistry and Molecular Pharmacology, New York University Grossman School of Medicine, New York, NY, United States; ^6^Department of Pathology, New York University Grossman School of Medicine, New York, NY, United States; ^7^Department of Psychiatry, New York University Grossman School of Medicine, New York, NY, United States

**Keywords:** Alzheimer's disease, epilepsy, choroid plexus, proteomics, laser capture microdissection

## Abstract

**Introduction:**

Alzheimer's disease (AD) and epilepsy are reciprocally related. Among sporadic AD patients, clinical seizures occur in 10–22% and subclinical epileptiform abnormalities occur in 22–54%. Cognitive deficits, especially short-term memory impairments, occur in most epilepsy patients. Common neurophysiological and molecular mechanisms occur in AD and epilepsy. The choroid plexus undergoes pathological changes in aging, AD, and epilepsy, including decreased CSF turnover, amyloid beta (Aβ), and tau accumulation due to impaired clearance and disrupted CSF amino acid homeostasis. This pathology may contribute to synaptic dysfunction in AD and epilepsy.

**Methods:**

We evaluated control (*n* = 8), severe AD (*n* = 8; A3, B3, C3 neuropathology), and epilepsy autopsy cases (*n* = 12) using laser capture microdissection (LCM) followed by label-free quantitative mass spectrometry on the choroid plexus adjacent to the hippocampus at the lateral geniculate nucleus level.

**Results:**

Proteomics identified 2,459 proteins in the choroid plexus. At a 5% false discovery rate (FDR), 616 proteins were differentially expressed in AD vs. control, 1 protein in epilepsy vs. control, and 438 proteins in AD vs. epilepsy. There was more variability in the epilepsy group across syndromes. The top 20 signaling pathways associated with differentially expressed proteins in AD vs. control included cell metabolism pathways; activated fatty acid beta-oxidation (*p* = 2.00 x 10^−7^, z = 3.00), and inhibited glycolysis (*p* = 1.00 x 10^−12^, z = −3.46). For AD vs. epilepsy, the altered pathways included cell metabolism pathways, activated complement system (*p* = 5.62 x 10^−5^, z = 2.00), and pathogen-induced cytokine storm (*p* = 2.19 x 10^−2^, z = 3.61). Of the 617 altered proteins in AD and epilepsy vs. controls, 497 (81%) were positively correlated (*p* < 0.0001, *R*^2^ = 0.27).

**Discussion:**

We found altered signaling pathways in the choroid plexus of severe AD cases and many correlated changes in the protein expression of cell metabolism pathways in AD and epilepsy cases. The shared molecular mechanisms should be investigated further to distinguish primary pathogenic changes from the secondary ones. These mechanisms could inform novel therapeutic strategies to prevent disease progression or restore normal function. A focus on dual-diagnosed AD/epilepsy cases, specific epilepsy syndromes, such as temporal lobe epilepsy, and changes across different severity levels in AD and epilepsy would add to our understanding.

## Introduction

Alzheimer's disease (AD) and epilepsy are reciprocally related: AD increases the risk of late-onset seizures, and epilepsy increases the risk of cognitive impairment (1–10), suggesting common molecular mechanisms. Seizures occur in 10–22% of sporadic AD (sAD) patients, subclinical epileptiform abnormalities in 22–54% of AD patients, (11–17) and cognitive deficits occur in up to 80% of epilepsy patients (1–3, 18). Non-convulsive seizures and subclinical electroencephalography (EEG) abnormalities are common and underrecognized in AD patients and may accelerate structural and cognitive disorders (4, 14, 15, 17, 19). In AD patients with epileptiform activity, the Mini-Mental State Examination (MMSE) score decreased faster compared to AD patients without epileptiform activity (15). Furthermore, anti-seizure medications [ASMs; e.g., levetiracetam (LEV)] decreased neuronal hyperexcitability and improved cognition in animal models and in patients with mild cognitive impairment (MCI) and are being investigated in ongoing studies for AD (20–24). Cognitive deficits are common in patients with chronic epilepsy, particularly in temporal lobe epilepsy (TLE), and late-onset epilepsy (8, 9, 18, 25, 26). Epilepsy patients had a faster MMSE decline than non-epilepsy patients (27), a 2-fold increased dementia risk when compared to controls (28), and a 3-fold increased dementia incidence in late-onset epilepsy when compared to non-epilepsy patients (9). Cognitive deficits and epileptiform activity are linked with amyloid beta (Aβ) and tau pathology in AD and epilepsy (3, 19, 25, 29, 30). Cognitive performance was impaired with altered cerebrospinal fluid (CSF) Aβ42 and EEG abnormalities in patients with late-onset epilepsy of unknown etiology and MCI when compared to MCI patients without epilepsy (26). Furthermore, some patients with late-onset epilepsy of unknown etiology develop pathogenic levels of AD biomarkers Aβ42 and tau that indicate an ongoing neurodegeneration process and a risk factor for AD (31). Compared to AD patients without seizures, those with seizures had increased Aβ and tau pathology via mTOR activation in the temporal cortex (32). An mTOR inhibitor improved cognition and ameliorated AD pathology in a 5xTg AD model (32), highlighting the therapeutic potential of exploring the pathways involved in the bidirectional relationship between AD and seizures.

The choroid plexus is impacted in both AD and epilepsy. It is the primary source for CSF production and is essential in the maintenance and function of the brain (33). This region undergoes age-related pathological changes (e.g., altered volume, epithelial atrophy, thickened basement membrane, and stroma fibrosis) that decrease CSF turnover (33–36). Aβ accumulation in the choroid plexus results from mitochondrial deficits, oxidative stress, and cytoskeletal dysregulation (34, 37–39). These pathogenic changes alter nutrient and ion secretion, impairing brain homeostasis (33, 35, 40). In epilepsy, choroid plexus and hippocampal inflammation occur ipsilateral to the seizure focus (41). CSF amino acid homeostasis is disrupted in epilepsy patients and animal epilepsy models (42–45).

We and others have identified AD protein changes in multiple brain regions over the disease course (46). These include glial proteins (47), Aβ, and tau levels that correlate with spliceosome activity (48–50), synaptic dysfunction (51, 52), and tau interacting proteins involved in ubiquitination and phagosome maturation (29, 53). In epilepsy, we identified protein changes associated with increased translation and decreased oxidative phosphorylation and synaptogenesis (54). The molecular mechanisms in the choroid plexus of AD and epilepsy are not well-understood. Limited proteomic studies in AD choroid plexus (55) and CSF revealed protein changes in CSF, indicating altered astrocyte/microglial and sugar metabolism (56), neuroinflammation, cerebrovascular dysfunction, and apoptosis (57, 58). There are no proteomics studies in human epilepsy choroid plexus. With most AD clinical trials failing (59–66) and drug-resistant epilepsy rates stable for decades (67, 68), proteomics approaches may reveal unbiased comprehensive datasets to identify shared druggable protein targets. Identifying these mechanisms can inform therapeutic strategies to improve network function, limit disease progression, and potentially reverse functional and pathological changes.

## Materials and methods

### Brain tissue

Specimens were acquired under protocols with Institutional Review Board (IRB) approval at NYU Grossman School of Medicine, including autopsy tissues from the North American SUDEP Registry (NASR) at NYU CEC, NYU ADRC, and NYU Center for Biospecimen Research and Development (CBRD)/Department of Pathology. For epilepsy cases (*n* = 12), the inclusion criteria were those cases with temporal lobe epilepsy or likely temporal lobe involved epilepsy as determined from the review of available medical records, as well as additional epilepsy cases that were age-matched to the other groups and enrolled in NASR. For AD cases (*n* = 8), the inclusion criteria were those cases with severe AD pathology as indicated by the neuropathology score A3B3C3 (69) and part of the NYU ADRC, which allowed for age matching to the other groups. Control cases (*n* = 8) were selected to include those cases with no known significant neurology or neuropathology. Cases were further selected to include those that were age-matched and with hippocampal sections available at the level of the lateral geniculate nucleus (LGN) with adjacent choroid plexus present. The sample size was informed by ours and other prior studies (47, 49, 52, 54, 56, 70, 71). Case history is summarized in Table 1 and detailed in Supplementary Table 1.

**Table 1:** Case history summary.

**Study group**	**n**	**Sex (M/F)**	**Age (years)**	**PMI (hours)**	**Brain weight (grams)**
Control	8	5/3	57.8 ± 6.1	59.1 ± 14.3	1249.0 ± 130.7
AD	8	2/6	72.6 ± 9.5	23.6 ± 22.5	1063.4 ± 102.3
Epilepsy	12	11/1	45.4 ± 14.3	35.8 ± 19.7	1392.9 ± 169.3

From the available case information. See Supplementary Table 1 for detailed case history. Mean is indicated for age, PMI, and brain weight ± standard deviation (mean ± SD).

### Laser capture microdissection

**Figure 1:**
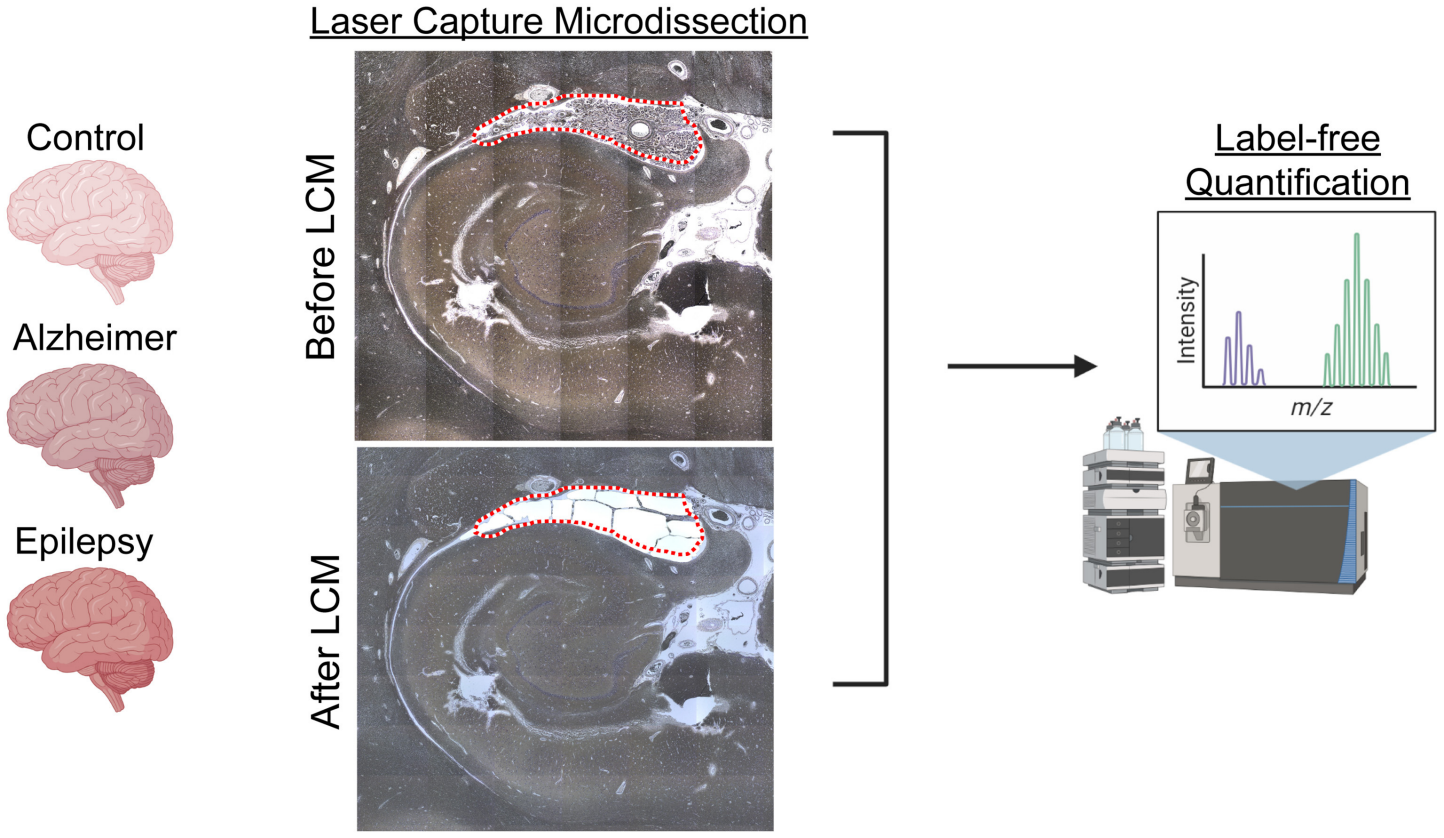
LCM and schematic approach overview. Choroid plexus (3 mm^2^), adjacent to the hippocampus at the level of LGN, was microdissected by LCM from FFPE autopsy brain tissue from control (*n* = 8), AD (*n* = 8), and epilepsy (*n* = 12) cases. Proteins were quantified by label-free quantitative mass spectrometry to identify protein differences.

Formalin-fixed, paraffin-embedded (FFPE) tissue was cut into 8 μg sections from autopsy hippocampal tissue at the level of the LGN with adjacent choroid plexus onto LCM PET membrane slides (54, 70, 72, 73) and stained with cresyl violet (74) for the localization of choroid plexus. Microdissected samples were collected at a consistent area per case of 3 mm^2^ into LC-MS grade water (Thermo Fisher Scientific) with the Leica LMD6500 LCM system. Samples were stored at −80°C until further processing. The schematic overview in Figure 1 was partially generated with Biorender.com.

### Label-free quantitative mass spectrometry LFQ-MS

#### Protein extraction and digestion

LCM-excised tissue samples were solubilized and digested using the SPEED sample prep workflow (75). In brief, tissue sections were incubated in 10 μl of LC-MS grade trifluoroacetic acid (TFA) for 5 min at 73°C. TFA was neutralized by 10x dilution (v:v) with 2M TRIS containing 10 mM Tris (2-carboxyethyl) phosphine TCEP and 20 mM chloroacetic acid (CAA) and incubated at 95°C for 10 min. For enzymatic digestion, samples were diluted 6x (v:v) with water containing 1 μg of sequencing-grade trypsin. Digestion was carried out at 37°C overnight and halted by acidification to 2% of TFA.

#### LC-MS/MS

LC separation was performed online on an Evosep One (Evosep) LC utilizing Dr. Maisch ReproSil-Pur 120 C18 AQ, 1.9-μm bead (150 μm ID, 15 cm long, cat# EV-1106) analytical column. Peptides were gradient eluted from the column directly into an Orbitrap HFX mass spectrometer using the 88-min extended Evosep method (SPD15) at a flow rate of 220 nl/min. The mass spectrometer was operated in data-independent acquisition (DIA) mode (76) acquiring MS/MS fragmentation across 22 m/z windows after every MS full-scan event.

High-resolution full MS spectra were acquired with a resolution of 120,000, an AGC target of 3e6, with a maximum ion injection time of 60 ms, and a scan range of 350 to 1650 m/z. Following each full MS scan, 22 data-independent HCD MS/MS scans were acquired at a resolution of 30,000, an AGC target of 3e6, and a stepped normalized collision energy (NCE) of 22.5, 25, and 27.5.

### Data analysis

MS data were analyzed using the Spectronaut^®^ software (https://biognosys.com/shop/spectronaut) and searched in direct DIA mode against the *homo sapiens* UniProt database (http://www.uniprot.org/). The database search used the integrated search engine Pulsar. For searching, enzyme specificity was set to trypsin with two or fewer missed cleavages. Oxidation of methionine was searched as a variable modification, and carbamidomethylation of cysteines was searched as a fixed modification. The false discovery rate (FDR) for peptide, protein, and site identification was set to 1%. Protein quantification was done on the MS/MS level using the three most intense fragment ions per precursor. Subsequent data analysis used Perseus (77) (http://www.perseus-framework.org/), R environment (http://www.r-project.org/), or Prism GraphPad for statistical computing and graphics. Raw data are available on the MassIVE server (https://massive.ucsd.edu/) under accession MSV000091370.

The protein expression matrix (*n* = 2,498) was filtered to remove the proteins that were non-human, common lab contaminants, and those proteins observed in less than half of all the three groups (*n* = 2,459). For principal component analysis (PCA), missing values were imputed from the normal distribution with a width of 0.3 and a downshift of 1.8 (relative to measured protein intensity distribution) in Perseus (77). Unpaired *t*-tests were performed in Perseus v. 1.6.2.3 (77) to detect significant changes in protein expression. A comparison of the significant proteins common to each pairwise comparison was evaluated by a Venn diagram generated from InteractiVenn (78). Cell-type annotations for each protein were evaluated in comparison to a reference choroid plexus dataset (79), as we have similarly done previously in other brain regions with enrichment evaluated by a Fisher's exact test (54, 70, 71, 73, 80, 81). The signaling pathways associated with the differentially expressed proteins were assessed by Ingenuity Pathway Analysis (IPA, Qiagen). All detected proteins were included in the dataset for each pairwise comparison, including the UniProtID, fold change, and *p*-value. Core analysis was performed in each brain region for proteins at an FDR of < 5%. Pathways were considered enriched at a *p*-value of overlap of < 0.05 and to be activated/inhibited as a result of combined protein fold changes in a pathway as reflected by a |z-score| of ≥2. Correlation analyses were performed by Pearson's correlation in GraphPad Prism. Data were also compared to previous AD studies and recently compiled in our NeuroPro database v1.12 (https://neuropro.biomedical.hosting/) (82). To identify basement membrane proteins (by cell component GO term), 616 proteins in AD vs. control were evaluated by STRING v11.5 (https://string-db.org/).

#### Immunohistochemistry

Immunohistochemistry was performed to validate the protein of interest, transmembrane protein 106B (TMEM106B) (52, 73, 83, 84). The FFPE sections (8 μm) were deparaffinized and rehydrated in a series of xylenes and ethanol dilutions. A heat-induced antigen retrieval was performed with 10 mM sodium citrate, 0.05% Triton-X 100; pH 6. Blocking with 10% normal donkey serum was followed by a TMEM106B primary antibody (1:100, Sigma HPA058342) and AQP1 (1:100, Santa Cruz sc-25287) overnight at 4°C. Sections were incubated with donkey anti-rabbit Alexa-Fluor 647 and Alexa-Fluor 488 secondary antibodies (1:500, Thermo Fisher Scientific, Invitrogen), counterstained with DAPI (Sigma D9542), and coverslipped.

Whole-slide scanning was performed at × 20 magnification with a Leica Aperio Versa 8 microscope using the same settings for each slide. There were three to four images at × 10 magnification collected for each case (*n* = 5 control, *n* = 5 AD, *n* = 5 epilepsy). Images were analyzed using Fiji ImageJ to compare the average amount of TMEM106B positive area among the groups. The same binary threshold was used for all images to determine the number of TMEM106B positive pixels in each image, which was reported as a percentage of the total image area. A Mann–Whitney U-test was performed for statistical analysis; a *p*-value of < 0.05 was considered significant.

## Results

### Protein differential expression

**Figure 2:**
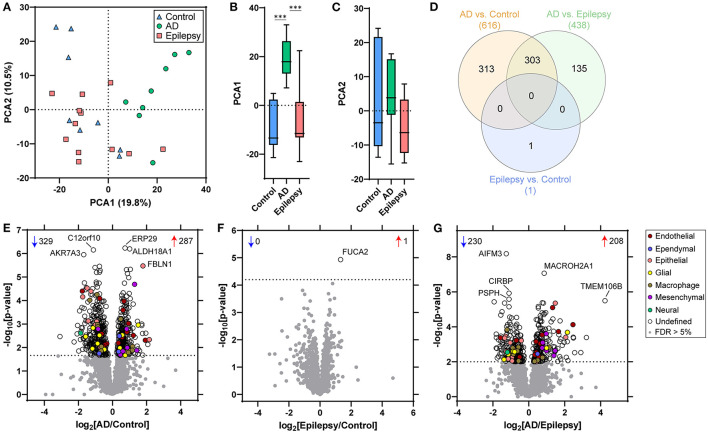
PCA and proteomic differences in the choroid plexus of control, epilepsy, and AD patients. **(A)** Principal component analysis (PCA) shows the distribution of control (*n* = 8), AD with A3, B3, C3 neuropathology (*n* = 8), and epilepsy (*n* = 12) for the 2,459 proteins detected in choroid plexus. **(B, C)** There is a segregation of AD from control (*p* < 0.0001) and epilepsy (*p* < 0.0001) in PCA1, but no segregation in PCA2 (one-way ANOVA with *post-hoc* Tukey's test). **(D)** Differential expression analysis for each pairwise comparison is indicated, as well as an overlap in the number of significant proteins, at a 5% false discovery rate (FDR; dotted line) when comparing **(E)** AD vs. control (616 proteins), **(F)** epilepsy vs. control (1 protein), and **(G)** AD vs. epilepsy (438 proteins). Annotations include the number of significantly increased (red arrows) and decreased (blue arrows) proteins. The top five altered proteins are annotated by gene name, and choroid plexus cell-type annotations for each significant protein are indicated.

Protein differential expression analysis was evaluated in control (*n* = 8), AD (*n* = 8), and epilepsy cases (*n* = 12) from the autopsy brain tissue with LFQ-MS in the microdissected choroid plexus (Table 1, Figure 1, Supplementary Table 1). LFQ-MS identified 2,459 proteins in the choroid plexus of the cases analyzed, detected in at least 50% of the cases in any of the groups. PCA showed significant segregation of AD cases from control (*p* < 0.0001) and epilepsy (*p* < 0.0001) cases in PCA1 (Figures 2A–C). There was more variability in the epilepsy group that included various syndromes. In addition to the disease group, sex contributed to some differences observed on the PCA (*p* = 0.023), while age did not (*p* = 0.89) as observed by a multiple variable linear regression analysis (Supplementary Table 2).

With an unpaired *t*-test followed by permutation-based FDR at 5%, there were significant differences between AD and control cases in 616 proteins, between epilepsy and control cases in 1 protein, and between AD and epilepsy cases in 438 proteins (Figures 2D–G, Supplementary Table 3). There were 303 proteins different in AD when compared to both control and epilepsy cases (Figure 2D). The top 20 most significant proteins altered in the AD vs. control and AD vs. epilepsy pairwise comparisons are summarized in Tables 2, 3. For epilepsy vs. control, the differentially expressed protein FUCA2 (alpha-L-fucosidase 2) was increased by 2.5-fold (*p* = 1.17 x 10^−5^). There were trending differences (*p* < 0.05, FDR >5%) in epilepsy vs. control for 216 proteins (Supplementary Table 3).

**Table 2:** Top 20 significant proteins in AD vs. control.

**Gene ID**	**Protein name**	**UniProt ID**	***p*-value**	**Fold change**
**Increased**				
ERP29	Endoplasmic reticulum resident protein 29	P30040	5.83E-07	1.7
ALDH18A1	Delta-1-pyrroline-5-carboxylate synthase	P54886	6.33E-07	2.0
FBLN1	Fibulin-1	P23142	3.38E-06	3.4
FAHD1	Acylpyruvase FAHD1, mitochondrial	Q6P587	4.73E-06	2.1
HIBADH	3-hydroxyisobutyrate dehydrogenase, mitochondrial	P31937	4.91E-06	1.8
NUCB2	Nucleobindin-2	P80303	7.16E-06	1.8
HADH	Hydroxyacyl-coenzyme A dehydrogenase, mitochondrial	Q16836	1.07E-05	1.9
LETM1	Mitochondrial proton/calcium exchanger protein	O95202	1.83E-05	1.6
FBN1	Fibrillin-1 [Cleaved into: Asprosin]	P35555	2.04E-05	2.5
**Decreased**				
C12orf10	UPF0160 protein MYG1, mitochondrial	Q9HB07	7.02E-07	2.2
AKR7A3	Aflatoxin B1 aldehyde reductase member 3	O95154	1.12E-06	3.2
DCTN2	Dynactin subunit 2	Q13561	3.50E-06	1.7
YWHAB	14-3-3 protein beta/alpha	P31946	3.94E-06	2.0
EIF3A	Eukaryotic translation initiation factor 3 subunit A	Q14152	5.28E-06	1.7
NAP1L4	Nucleosome assembly protein 1-like 4	Q99733	6.21E-06	1.7
AKR7A2	Aflatoxin B1 aldehyde reductase member 2	O43488	6.36E-06	1.9
EZR	Ezrin	P15311	1.65E-05	1.9
ALDOA	Fructose-bisphosphate aldolase A	P04075	1.78E-05	3.1
PPM1B	Protein phosphatase 1B	O75688	1.82E-05	1.7
RDX	Radixin	P35241	1.97E-05	1.9

**Table 3:** Top 20 significant proteins in AD vs. epilepsy.

**Gene ID**	**Protein name**	**UniProt ID**	***p*-value**	**Fold change**
**Increased**				
MACROH2A1	Core histone macro-H2A.1	O75367	8.64E-08	1.8
TMEM106B	Transmembrane protein 106B	Q9NUM4	3.22E-06	18.9
ERLIN2	Erlin-2	O94905	4.23E-06	1.7
HIBADH	3-hydroxyisobutyrate dehydrogenase, mitochondrial	P31937	4.35E-06	1.8
FBLN1	Fibulin-1	P23142	4.43E-06	2.8
VAPA	Vesicle-associated membrane protein-associated protein A	Q9P0L0	6.84E-06	1.4
TGM2	Protein-glutamine gamma-glutamyltransferase 2	P21980	8.01E-06	2.5
XRCC5	X-ray repair cross-complementing protein 5	P13010	8.42E-06	1.4
ATP5PD	ATP synthase subunit d, mitochondrial	O75947	1.54E-05	1.9
FAHD1	Acylpyruvase FAHD1, mitochondrial	Q6P587	1.56E-05	1.7
PNPLA6	Patatin-like phospholipase domain-containing protein 6	Q8IY17	1.74E-05	1.8
NUCB2	Nucleobindin-2	P80303	1.98E-05	1.8
**Decreased**				
AIFM3	Apoptosis-inducing factor 3	Q96NN9	6.58E-09	2.4
CIRBP	Cold-inducible RNA-binding protein	Q14011	1.19E-06	2.2
PSPH	Phosphoserine phosphatase	P78330	2.63E-06	2.1
MPI	Mannose-6-phosphate isomerase	P34949	3.70E-06	3.8
KCNJ13	Inward rectifier potassium channel 13	O60928	4.40E-06	2.6
SLC39A12	Zinc transporter ZIP12	Q504Y0	7.08E-06	2.1
AKR7A3	Aflatoxin B1 aldehyde reductase member 3	O95154	1.36E-05	2.8
GLUL	Glutamine synthetase	P15104	2.12E-05	3.0

**Figure 3:**
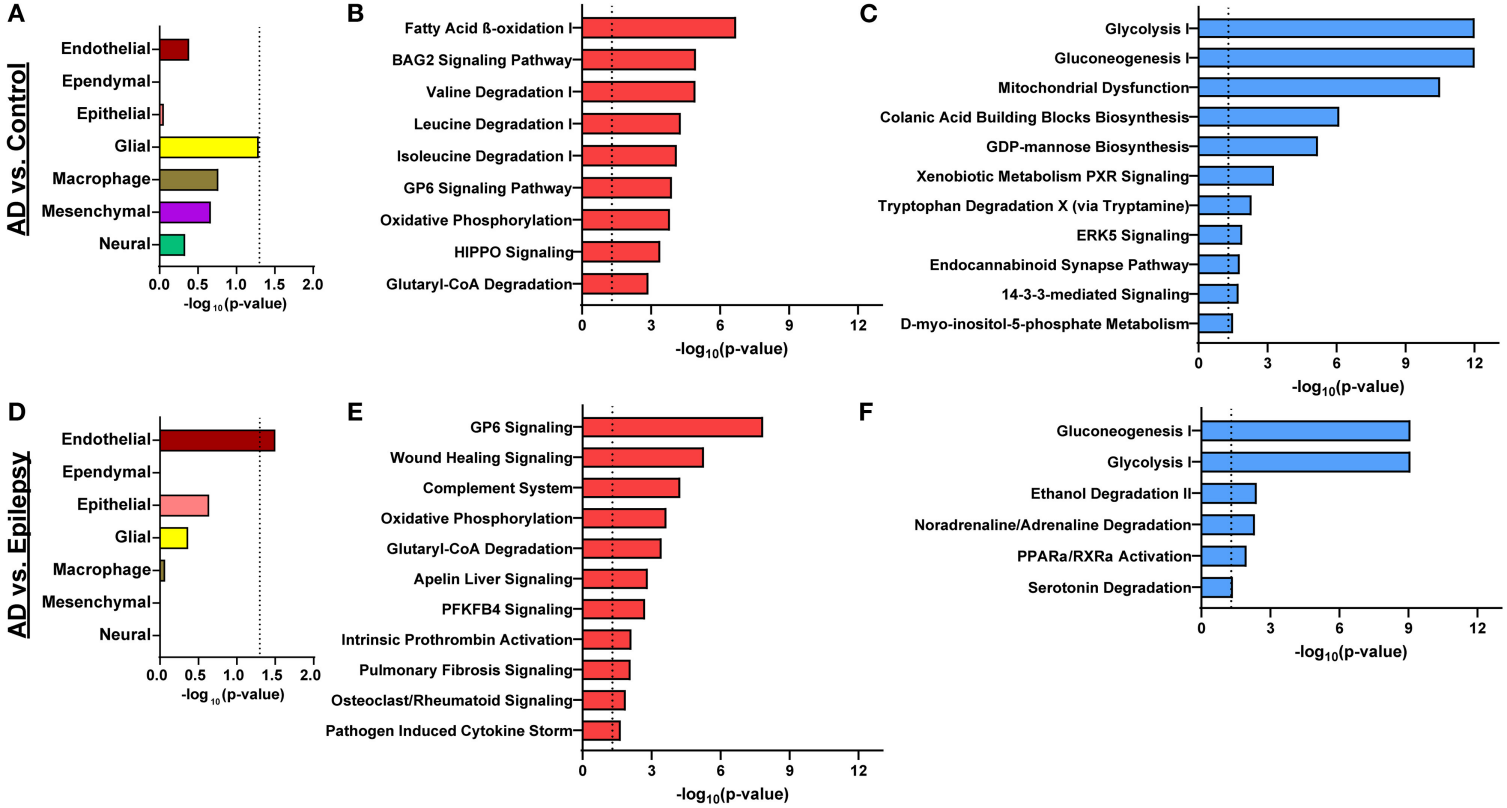
Cell-type enrichment and signaling pathways associated with proteomics differences. **(A)** Cell-type annotation analysis of differentially expressed proteins in AD vs. control by Fisher's exact test indicates a trend in enrichment (*p* = 0.051) for glial proteins. **(B, C)** For AD vs. control, the 616 differentially expressed proteins are significantly associated with 9 activated pathways (red) and 11 inhibited pathways (blue; *p*-value of overla*p* < 0.05, z-score ≥ |2|). **(D)** Cell-type enrichment analysis for differentially expressed proteins in AD vs. epilepsy indicates enrichment for endothelial cell proteins (*p* = 0.031). **(E, F)** For AD vs. epilepsy, the 438 differentially expressed proteins are significantly associated with 11 activated pathways and 6 inhibited pathways. The dotted lines indicate *p* = 0.05.

After cell-type annotation of proteins, most proteins were “undefined” and likely expressed by multiple cell types, or their association is unknown (Figures 2, 3, Supplementary Table 3). After “undefined,” the most abundant annotation for significant proteins was for endothelial proteins (2.4%, 15 proteins) in AD vs. control and both endothelial and epithelial proteins (3.2%, 14 proteins each) in AD vs. epilepsy. Cell-type enrichment analysis (Fisher's exact test) indicated that glial proteins (1.9%, 12 proteins) were trending in enrichment (*p* = 0.051) in AD vs. control, and endothelial proteins were enriched (*p* = 0.031) in AD vs. epilepsy (Figure 3).

### Pathway analysis

**Figure 4:**
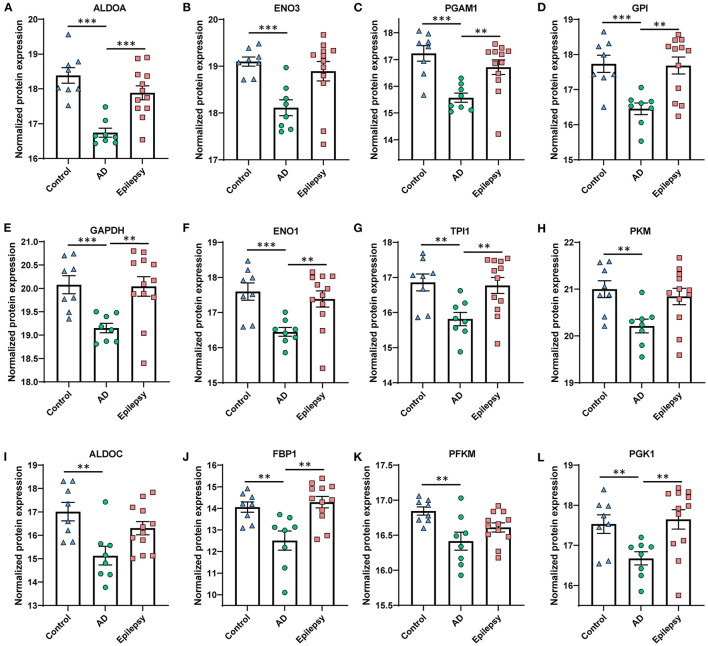
Proteins in AD vs. control associated with the top altered pathway, glycolysis inhibition. The glycolysis signaling pathway was the most significantly altered and was the most affected by the fold change of proteins in AD vs. control (*p* = 1.00 x 10^−12^, z = −3.46). **(A–L)** The proteins are depicted by order of decreasing significance. Those proteins that are significant at 5% FDR are indicated for all pairwise comparisons, with the *p*-values as indicated. ***p < 0.001, **p < 0.01. Error bars indicate SEM.

In AD vs. control (Figures 3B, C), pathway analysis of the significantly altered proteins identified 142 signaling pathways associated with the 616 proteins (*p*-value of overlap < 0.05); 20 of these pathways were significantly impacted by fold change as reflected by the z-score (|z| ≥ 2; Supplementary Table 4). Top signaling pathways were associated with cell metabolism, including activated fatty acid beta-oxidation (*p* = 2.00 x 10^−7^, z = 3.00) and inhibited glycolysis (*p* = 1.00 x 10^−12^, z = −3.46; Figure 4). Three branched-chain amino acid degradation pathways were activated: valine degradation I (*p* = 1.17 x 10^−5^, z = 2.45), leucine degradation I (*p* = 5.13 x 10^−5^, z = 2.00), and isoleucine degradation I (*p* = 7.59 x 10^−5^, z = 2.24). There was BAG2 signaling activation (*p* = 1.12 x 10^−5^, z = 2.00) with several decreased proteasome proteins, as well as 14-3-3-mediated signaling inhibition (*p* = 1.82 x 10^−2^, z = −2.12).

In AD vs. epilepsy (Figures 3E, F), pathway analysis of the significantly altered proteins identified 137 signaling pathways associated with the 438 proteins (*p*-value of overlap < 0.05) and 17 pathways were significantly impacted by fold change as reflected by the z-score (|z| ≥ 2; Supplementary Table 5). The top 20 signaling pathways similar to AD vs. control included five pathways associated with cell metabolism (gluconeogenesis I, glycolysis I, oxidative phosphorylation, and glutaryl-CoA degradation) and the GP6 signaling pathway that is related to platelet activation and thrombus formation. Unique to AD vs. epilepsy, there were two activated inflammation signaling pathways: complement system (*p* = 5.62 x 10^−5^, z = 2.00) and pathogen-induced cytokine storm (*p* = 2.19 x 10^−2^, z = 3.61).

In epilepsy vs. control, there were no pathways associated with the one altered protein FUCA2. Pathways associated with the 216 trending proteins at a *p*-value of < 0.05 with an FDR of >5% are detailed in Supplementary Table 6.

### TMEM106B validation and localization

**Figure 5:**
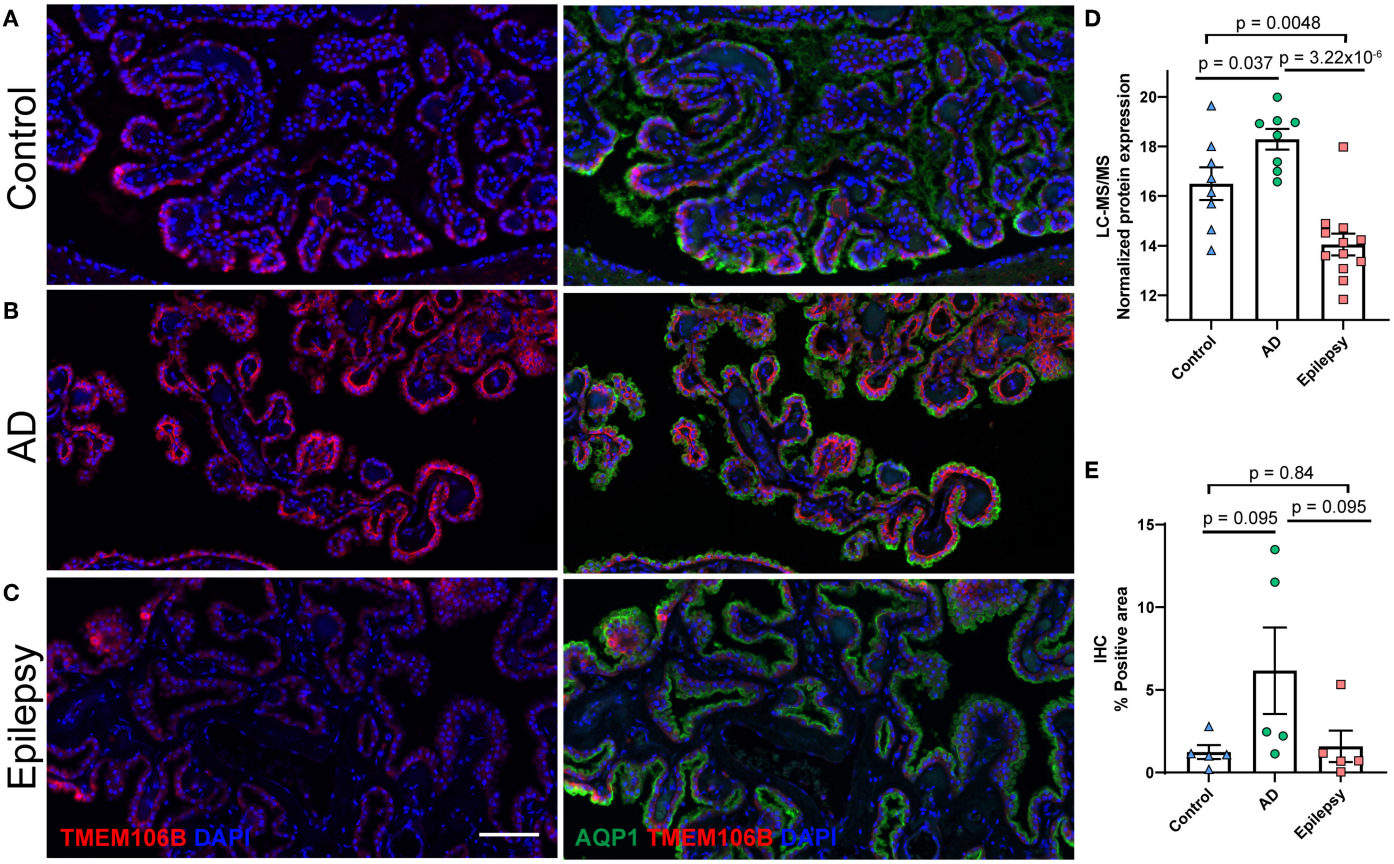
Protein candidate TMEM106B histological localization and quantification. Representative images from the **(A)** control, **(B)** AD, and **(C)** epilepsy groups of TMEM106B (red) localized in the basal membrane and epithelial marker AQP1 (green) in the apical membrane of epithelial cells of the choroid plexus adjacent to the hippocampus at the level of LGN. **(D)** TMEM106B quantification by LFQ-MS in control (*n* = 8), AD (*n* = 8), and epilepsy cases (*n* = 12). As determined by Student's two-tailed *t*-test with permutation correction at a 5% FDR, for AD vs. epilepsy, there was an 18.9-fold increase (p = 3.22 x 10^−6^, FDR <5%), for AD vs. control, there was a 3.5-fold increase (*p* = 0.037, FDR >5%), and for epilepsy vs. control, there was a 5.5-fold decrease (*p* = 0.0048, FDR >5%). **(E)** Immunohistochemistry from five cases/group shows using semiquantitative analysis that TMEM106B expression follows a similar trend observed in LFQ-MS, AD vs. epilepsy (3.9-fold increase, *p* = 0.095), AD vs. control (5.0-fold increase, *p* = 0.095), and epilepsy vs. control (1.3-fold increase, *p* = 0.84) by the Mann–Whitney U-test. Scale bar 100 um. Error bars indicate SEM.

TMEM106B (Q9NUM4) was among the top 20 most significantly altered proteins when comparing AD vs. epilepsy (Table 3) with the highest fold change at an 18.9-fold increase (*p* = 3.22 x 10^−6^) and was a top protein candidate for validation with cell and regional localization. For AD vs. control by LFQ-MS, there was a 3.5-fold increase (*p* = 0.04, not significant at 5% FDR). By immunohistochemistry, TMEM106B was predominantly localized in epithelial cells at the basal membrane (Figure 5). The epithelial cell marker in the choroid plexus, aquaporin 1 (AQP1), was evaluated for colocalization and was present in the apical membrane of epithelial cells. Validation of the LFQ-MS findings in five cases per group with the semiquantification of immunohistochemistry similarly showed the same trends for TMEM106B, with a 3.9-fold increase in AD vs. epilepsy (*p* = 0.095) and a 5.0-fold increase in AD vs. control (*p* = 0.095).

### AD and epilepsy correlation analysis

**Figure 6:**
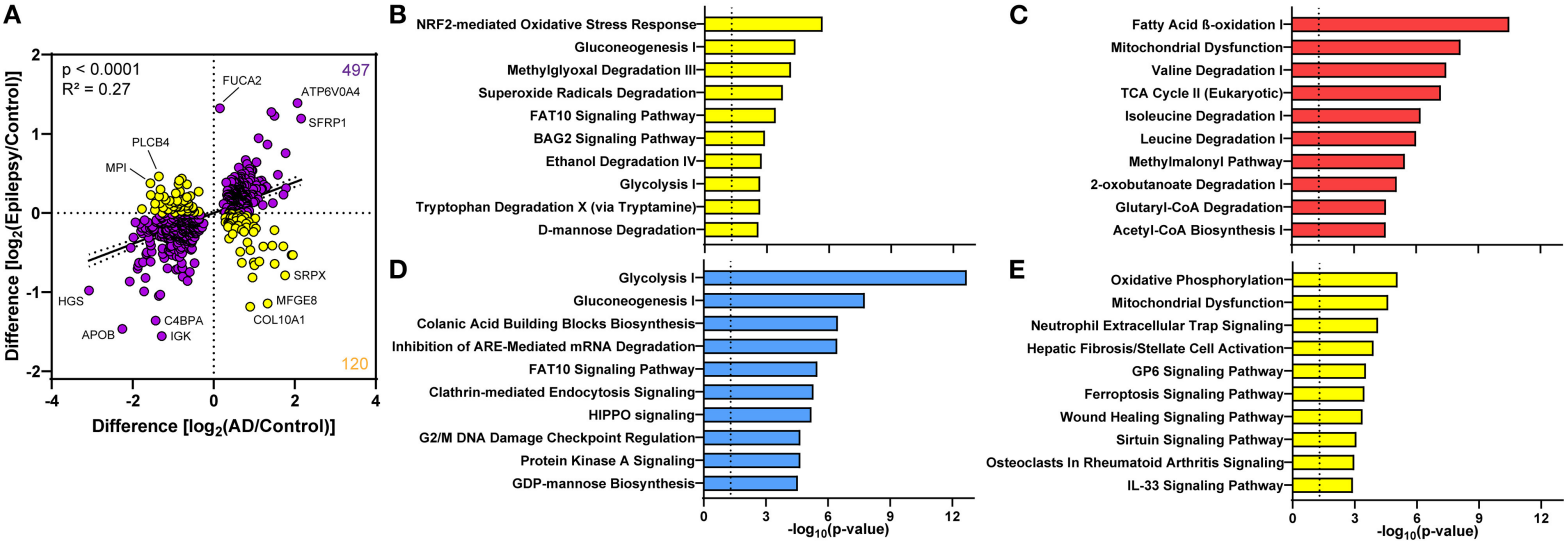
Proteomic differences in the choroid plexus of AD and epilepsy cases positively correlate. **(A)** Of the 617 altered proteins in AD and epilepsy cases when compared to controls, 497/617 (81%) changed in the same direction (purple) and 120/617 (19%) in the opposite direction (yellow) with an overall positive correlation (*p* < 0.0001, *R*^2^ = 0.27). Several of the proteins with the highest fold change are annotated by the gene name. The top 10 signaling pathways associated with the proteins in each quadrant from the correlation show those pathways **(B)** down in AD and up in epilepsy, **(C)** up in both AD and epilepsy, **(D)** down in both AD and epilepsy, and **(E)** up in AD and down in epilepsy.

Although few proteomic differences in epilepsy vs. control reached the 5% FDR, 617 proteins altered in AD and epilepsy vs. controls had a positive correlation in expression levels (*p* < 0.0001, *R*^2^ = 0.27, Figure 6A). There were 81% (497/617) of proteins changing in the same direction and 19% (120/617) of proteins changing in the opposite direction, indicating that many protein changes in AD also trend in epilepsy cases but do not reach significance in these cohorts. The top 10 pathways associated with these proteins were specified by those up in both disease groups, down in both, or changing in the opposite direction (Figures 6B–E, Supplementary Tables 7–10).

### Comparison to other AD studies

We compared the choroid plexus protein differences in AD vs. control to AD-related proteomics studies in our NeuroPro database (82) that compiles results from 38 other proteomics studies, with multiple brain regions, subtypes of disease progression, and types of pathology. There was an overlap of the identified proteins from the choroid plexus with 525 confirmed from previous studies and 91 unique proteins via proteomics to the choroid plexus (Supplementary Tables 11, 12). Of the 525 confirmed proteins, 114 proteins were altered in AD when compared to controls from 9 other brain regions in previous studies. Among the 91 unique proteins by proteomics to choroid plexus, there were several increased collagen and aldehyde dehydrogenase proteins.

## Discussion

We identified protein differences in the choroid plexus of AD cases with severe neuropathology when compared to control and epilepsy cases, with top significant pathways related to activated fatty acid beta-oxidation and inhibited glycolysis. The protein differences in the AD group correlated with the same trends in epilepsy when compared to control cases, with more variability in the epilepsy group.

### AD vs. control

We identified pathways associated with altered cell energy metabolism indicating a shift from glucose-mediated energy production to fatty acid beta-oxidation activation and glycolysis inhibition, coupled with activated branched-chain amino acid degradation. This shift was further reflected by trends in ketogenic pathways, with mild activation of ketolysis (*p* = 8.41 x 10^−5^, z = 1.00) and ketogenesis (*p* = 1.29 x 10^−4^, z = 1.00). There was oxidative phosphorylation activation, with many increased proteins in complex I (NDUF proteins), as well as complexes II and V. The elevated abundance of these mitochondrial proteins may indicate increased expression or mitochondrial biogenesis that occurs with ketosis (85). Brain imaging studies found hypometabolism in AD patients consistent with low glucose in some brain regions (86). We detected the glucose transporter GLUT1 (SLC2A1) (87) altered in some cells in an AD mouse model (88), but this was not different from controls in the choroid plexus. Future studies should evaluate this further in specific choroid plexus cell types and correlate with neighboring brain tissues and CSF protein levels, as well as clinical variables such as disease progression. Evaluating how these altered pathways may impact ketosis induction may provide insights into the mechanisms of cognitive dysfunction and resilience (89–92).

Other altered pathways associated with AD include BAG2 and 14-3-3 signaling. In the current study, BAG2 signaling activation included nine decreased proteasome proteins and two increased heat shock proteins. This pathway is associated with multiple functions such as cytoskeleton maintenance, including proteasome-independent phosphorylated tau degradation (93). We detected total tau (MAPT) in most cases (*n* = 7 control, *n* = 2 AD, *n* = 9 epilepsy), but this was not different among the groups. Regarding proteasome proteins, we detected a number of these proteins, but those that were significant were all decreased and associated with this pathway. Previous studies have shown that proteasome proteins tend to be increased in AD when compared to controls in other brain regions when searched in our NeuroPro database (82). Follow-up studies should evaluate this finding in choroid plexus to determine whether these decreased proteins are associated with the dysfunction of protein clearance, altered in specific cell types, or present in another insoluble fraction for example. Additionally, 14-3-3-mediated signaling was inhibited with decreased 14-3-3 proteins (YWHAB, YWHAE, YWHAG, YWHAQ, and YWHAZ). The proteins in this pathway are also associated with multiple cellular functions, and in AD, they colocalize with neurofibrillary tau tangles and are increased in CSF, with correlations to clinical variables (94, 95). Evidence suggests that 14-3-3 proteins are decreased in the frontal cortex tissue, as well as in some studies from our NeuroPro database in most brain regions and in a limited choroid plexus proteomics study (55, 82, 96).

Proteomics analyses in human AD choroid plexus have been limited to less sensitive approaches (55), and transcriptomic studies have been limited to two RNA microarray analyses (97, 98). In the first RNA microarray study, choroid plexus epithelial cells were microdissected from AD and controls with differences related to increased oxidative stress and protein ubiquitin pathways and decreased glutathione-mediated detoxification and urea cycle pathways (99). In the second RNA microarray study, bulk AD choroid plexus were compared to controls with differences related to upregulated metabolic and immune-related pathways and downregulated methionine degradation and protein translation (98). We identified trends in these signaling pathways (*p*-value of overlap < 0.05, z-score n.s.), including mTOR signaling (98), methionine degradation pathways (98), unfolded protein response (99), protein ubiquitination pathway, (99) urea cycle, (99) and glutathione-mediated detoxification (99). In contrast to previous studies, NRF2 oxidative stress (99) and aldosterone signaling in epithelial cells (99) trended down.

Other altered proteins in aging or AD choroid plexus were identified by non-proteomic studies (33, 35, 100), including basement membrane thickening, decreased clusterin, TTR, LRP2, IGF1, and gelsolin, and increased LRP1 and PGP. We identified 17 proteins associated with the basement membrane (GO cellular component GO:0005604) that were all increased and may be consistent with basement membrane thickening. Clusterin (CLU, also known as APOJ; P10909), an extracellular chaperone that traffics multiple proteins including Aβ in addition to other functions (100), was increased by 2.3-fold (*p* = 1.42 x 10^−4^). LRP1 was detected but not different. LRP2, TTR, PGP, gelsolin, and IGF1 were not detected.

We expected some similarities of proteins when comparing the choroid plexus to other studies evaluating CSF and blood vessel protein expression levels, as the choroid plexus produces CSF and also contains blood vessels. CSF proteomics analyses had identified altered metabolism proteins in AD vs. controls, some differing from the brain tissue (56, 101). Increased glycolysis proteins were identified in CSF, including a top candidate aldolase fructose-bisphosphate A (ALDOA) (101). Whereas, we identified a significant 3.1-fold decrease (*p* = 1.78 x 10^−5^) in ALDOA in the choroid plexus of AD. In a proteomics analysis of Aβ accumulation in blood vessels of cases with cerebral amyloid angiopathy (CAA) in the occipital/parietal lobes, one of the top altered proteins was high-temperature requirement serine peptidase 1 (HTRA1) which is suggested to remove misfolded or mislocalized peptides in an ATP-independent manner (102). From our NeuroPro database, this protein is also increased in a number of other studies in AD from various brain regions (82). Similarly, we identified a 2.8-fold increase in HTRA1 (*p* = 1.11 x 10^−3^).

### Epilepsy vs. control

In the epilepsy vs. control comparison, only one protein (FUCA2) was elevated with many trending proteins in this heterogeneous disease group. In the 216 proteins trending in epilepsy, FUCA1 was also increased and has a similar function to FUCA2 adding fucose to glycoproteins and can be associated with cell migration as suggested from elevation in various tumor types (103). From the pathways associated with trending proteins, there were similarities to those observed in AD when compared to controls that included fatty beta-oxidation and 14-3-3 signaling. There have been no related proteomics or transcriptomics studies in human epilepsy choroid plexus for comparison. It will be of interest in future studies to evaluate larger homogeneous cohorts to identify whether there are additional protein differences, as well as comparison to other AD groups with more mild pathology and AD cases with an epilepsy diagnosis.

### AD vs. epilepsy

In the AD vs. epilepsy comparison, most of the protein differences were also found when comparing AD to controls, and so many of the same signaling pathways were identified. Additionally, there was the activation of inflammatory-related pathways such as complement system and pathogen-induced cytokine storm that were associated with a number of complement and collagen proteins. Although there were many differences, the changes in AD also correlated with trends in epilepsy when compared to controls.

TMEM106B was a top protein candidate that was elevated in AD when compared to the epilepsy group. TMEM106B is a type II transmembrane protein that localizes to late endosomes and lysosomes in many cell types, including in both neurons and oligodendrocytes (104). Previous studies have shown that TMEM106B can fibrilize in a similar way as Aβ in AD and that TMEM106B filaments may form in an age-dependent manner (105–107). There was a similar trend for expression levels on LFQ-MS and histology, with differences related to the detection method (i.e., sensitivity and normalization).

The correlation of AD and epilepsy to controls from those proteins significant in at least one pairwise comparison identified a positive correlation, with the majority of proteins changing in the same direction. With these similar trends, as expected, many of the same signaling pathways were identified and were associated with a shift in cellular energy production. Among the top correlated proteins with the highest fold changes, there was increased ATP6V0A4 and decreased APOB. ATP6V0A4 is a vacuolar ATPase (108) and can be involved in several signaling pathways, including those associated with endocytosis. The top pathway associated with ATP6V0A4 (increased by 4.2-fold in AD and by 2.6-fold in epilepsy compared to controls) from the increased proteins in the correlation was the iron homeostasis signaling pathway (*p* = 3.80 x 10^−4^). APOB is an apolipoprotein that transports lipids in plasma and CSF (109) and is also involved in several signaling pathways including endocytosis. APOB is increased in AD CSF and plasma. (109) It is unclear whether these cases have lower APOB levels relative to the many controls with atherosclerosis (110) that were observed on neuropathology and whether these levels reflect those in the adjacent brain tissue or CSF. Some of the top protein differences between AD and epilepsy with the highest fold change from the correlation included increased MFGE8 (milk fat globule EGF and Factor V/VIII domain containing) by 2.5-fold in AD and decreased by 2.2-fold in epilepsy. An increase in AD may be expected as MFGE8 vascular deposition increases with age and it can interact with Aβ (111). As noted above, it will be of interest to evaluate these protein differences further in larger homogeneous epilepsy cohorts, as well as across the AD and epilepsy spectrums of disease. Furthermore, future mechanistic studies will be essential to elucidate the implications of these protein differences, i.e., how the altered signaling pathways directly or indirectly impact CSF production, turnover, and content.

### Limitations

Our study had limitations, including a small sample size. Our technique is less sensitive in detecting large membrane proteins, insoluble proteins, and low-abundance proteins (i.e., TTR, AQP1, and APP were not detected). Among the AD and epilepsy disease groups, heterogeneous clinical variables warrant further evaluation in future studies with larger samples, as do genetic risk factors (e.g., APOE, MTOR, APP, and PSEN1). Differences we identified in bulk choroid plexus should be explored with regard to specific cell types.

## Conclusion

We identified a shift in cell energy metabolism in the choroid plexus of AD patients with severe neuropathology and similar trends in epilepsy patients. Follow-up studies should evaluate the spectrum of AD and epilepsy, including those cases with dual diagnoses to identify potential molecular drivers of epilepsy and AD. This could empower novel and targeted therapies.

## Data availability statement

The datasets presented in this study can be found in online repositories. The names of the repository/repositories and accession number(s) can be found in the article/Supplementary material.

## Ethics statement

The studies involving human participants were reviewed and approved by NYU Grossman School of Medicine Institutional Review Board (IRB). The patients/participants provided their written informed consent to participate in this study.

## Author contributions

TW, OD, and DL contributed to the conception and design of the manuscript. DL, EK, AF, MT, DF, SD, and BU contributed to data collection. DL and EK performed data analyses. DL wrote the first draft of the manuscript. All authors contributed to the manuscript revision, read, and approved the submitted version.
